# *Fonsecaea pedrosoi* Sclerotic Cells: Secretion of Aspartic-Type Peptidase and Susceptibility to Peptidase Inhibitors

**DOI:** 10.3389/fmicb.2018.01383

**Published:** 2018-06-29

**Authors:** Vanila F. Palmeira, Fatima R. V. Goulart, Marcela Q. Granato, Daniela S. Alviano, Celuta S. Alviano, Lucimar F. Kneipp, André L. S. Santos

**Affiliations:** ^1^Laboratório de Estudos Avançados de Microrganismos Emergentes e Resistentes, Departamento de Microbiologia Geral, Instituto de Microbiologia Paulo de Góes, Universidade Federal do Rio de Janeiro, Rio de Janeiro, Brazil; ^2^Laboratório de Estrutura de Microrganismos, Departamento de Microbiologia Geral, Instituto de Microbiologia Paulo de Góes, Universidade Federal do Rio de Janeiro, Rio de Janeiro, Brazil; ^3^Laboratório de Taxonomia, Bioquímica e Bioprospecção de Fungos, Instituto Oswaldo Cruz, Fundação Oswaldo Cruz, Rio de Janeiro, Brazil; ^4^Programa de Pós-Graduação em Bioquímica, Instituto de Química, Universidade Federal do Rio de Janeiro, Rio de Janeiro, Brazil

**Keywords:** *Fonsecaea pedrosoi*, chromoblastomycosis, aspartic peptidase, peptidase inhibitors, antifungal drug

## Abstract

*Fonsecaea pedrosoi* is a dematiaceous fungus and the main causative agent of chromoblastomycosis that is a chronic disease usually affecting the human skin and subcutaneous tissues, which causes deformations and incapacities, being frequently refractory to available therapies. A typical globe-shaped, multiseptated and pigmented cells, known as sclerotic cells, are found in the lesions of infected individuals. In the present work, we have investigated the production of aspartic-type peptidase in *F. pedrosoi* sclerotic cells as well as the effect of peptidase inhibitors (PIs) on its enzymatic activity and viability. Our data showed that sclerotic cells are able to secrete pepstatin A-sensible aspartic peptidase when grown under chemically defined conditions. In addition, aspartic PIs (ritonavir, nelfinavir, indinavir, and saquinavir), which are clinically used in the HIV chemotherapy, significantly decreased the fungal peptidase activity, varying from 55 to 99%. Moreover, sclerotic cell-derived aspartic peptidase hydrolyzed human albumin, an important serum protein, as well as laminin, an extracellular matrix component, but not immunoglobulin G and fibronectin. It is well-known that aspartic peptidases play important physiological roles in fungal cells. With this task in mind, the effect of pepstatin A, a classical aspartic peptidase inhibitor, on the *F. pedrosoi* proliferation was evaluated. Pepstatin A inhibited the fungal viability in both cellular density- and drug-concentration manners. Moreover, HIV-PIs at 10 μM powerfully inhibited the viability (>65%) of *F. pedrosoi* sclerotic cells. The detection of aspartic peptidase produced by sclerotic cells, the parasitic form of *F. pedrosoi*, may contribute to reveal new virulence markers and potential targets for chromoblastomycosis therapy.

## Introduction

*Fonsecaea pedrosoi* is a melanized saprophytic filamentous fungus able to cause a chronic, progressive and granulomatous skin and/or subcutaneous tissue infections, named chromoblastomycosis, which occur most frequently in humid tropical and subtropical regions of America, Asia and Africa (Santos et al., 2007; Queiroz-Telles et al., 2017). This dimorphic fungus produce different morphotypes including conidia (reproduction structures) and mycelia (filamentous forms), both are usually found in its saprophytic lifestyle, as well as sclerotic cells (synonymous with muriform or medlar bodies), which are the parasitic forms observed in the infected tissues (Rippon, 1988). These brownish-yellow fungal cells with thick-pigmented walls are a hallmark in the histopathological diagnosis of chromoblastomycosis (Krzyściak et al., 2014). The morphology of sclerotic cell is well-known, but its physiology remains poorly studied, mainly because this tissue form is very hard to be induced *in vitro* and is not usually obtained in high quantities in its disarticulated state (Santos et al., 2007). Even though, there are several reports showing different procedures to induce *in vitro* sclerotic cell formation from chromoblastomycosis fungi, such as pH reduction, manganese deprivation, calcium or propranolol supplementation and natural culture medium formulated from tree fruits (Alviano et al., 1992; Mendoza et al., 1993; Silva et al., 2002, 2008). Studies conducted by our group revealed that sclerotic cells obtained *in vitro* were similar to those observed *in vivo*. The cellular morphology, ultrastructure, as well as the antigenic cross-reactivity between *in vivo* and *in vitro* sclerotic cells confirmed their similarity, showing that the latter can be used in experiments aiming to understand the physiopathology of chromoblastomycosis fungi (Silva et al., 2002).

In the past, sclerotic cells were mainly known as resistant forms able to survive inside the host tissues. However, several studies have shown that sclerotic cells are active parasitic forms involved directly with *F. pedrosoi* pathogenicity (Silva et al., 2002; Alviano et al., 2004; Siqueira et al., 2017). In addition, sclerotic cells are extremely resistant to immune system attack. Dong et al. (2014) reported a chitin-like component, expressed on the surface of *F. pedrosoi* sclerotic cells, able to inhibit dectin-1-mediated murine Th17 development by masking fungal β-glucans, which consequently blocks the recruitment of neutrophils to the infectious foci. Recently, chromoblastomycosis murine model studies revealed that only sclerotic cells depend on dectin-1 recognition to be internalized, suggesting different *F. pedrosoi*-host interaction strategies related to fungal morphotypes (Siqueira et al., 2017). Furthermore, those authors also reported that *F. pedrosoi* sclerotic cells are the ones responsible for the intense inflammatory reaction, correlated with the fungus persistence in the host, which leads to the chronic phase of chromoblastomycosis. These reasons could explain the difficulty in treating this chronic disease, even more after considering the fact that highly melanized sclerotic cells make the fungi much more resistant to different classes of antifungal drugs (Revankar and Sutton, 2010; Queiroz-Telles et al., 2017).

The chromoblastomycosis pathogenicity mechanisms are not well established. However, in recent years, our research group has described some enzymes involved in the physiopathology of chromoblastomycosis fungi, including peptidases (Kneipp et al., 2003, 2004, 2012; Palmeira et al., 2006a,b, 2008, 2017; Granato et al., 2015). It is known that proteolytic enzymes participate in infectious processes caused by a number of human pathogenic fungi, being main actors in several aspects of fungi-host interplays such as adhesion, invasion, nutrition, escape, proliferation, and differentiation (Monod et al., 2002; Yike, 2011; Puri et al., 2012). Over the last years, we identified and characterized the proteolytic activity secreted by *F. pedrosoi* conidia and mycelia which are correlated to important events such as cellular differentiation, growth and interaction with host cells (Palmeira et al., 2006a,b, 2008, 2017). Several studies have proposed that fungal peptidases are potential targets to develop new antifungal drugs (Pozio and Morales, 2005; Santos, 2010; Santos and Braga-Silva, 2013; Santos et al., 2013). Corroborating this statement, HIV aspartic peptidase inhibitors (HIV-PIs) are able to block the hydrolytic activity of aspartic peptidases released by *F. pedrosoi* conidial and mycelial forms as well as their *in vitro* growth (Palmeira et al., 2006b, 2008, 2017). Furthermore, HIV-PIs treatment restrained the conidia-into-mycelia differentiation as well as reduced their adhesion to mammalian cells (Palmeira et al., 2008).

For all the reasons elucidated above, in the present work, we have investigated the capability of *F. pedrosoi* sclerotic cells in releasing aspartic-type peptidase into the extracellular surrounding. Also, the effects of aspartic PIs were evaluated on fungal enzymatic activity and viability.

## Materials and Methods

### Chemicals

Saquinavir and nelfinavir were obtained from Hoffmann-La Roche AG (Grenzach-Wyhlen, Germany), indinavir from Merck Sharp & Dohme GmbH (Haar, Germany) and ritonavir from Abbot Park (Illinois, United States). The HIV-PIs were dissolved in absolute methanol to obtain a final concentration of 20 mM and stored at -20°C before use. Human serum albumin (HSA), bovine serum albumin (BSA), immunoglobulin G (IgG), laminin (LAM), fibronectin (FBN), *trans*-epoxysuccinyl L-leucylamido-(4-guanidino) butane (E-64), phenylmethylsulfonyl fluoride (PMSF), pepstatin A and 1,10-phenanthroline were purchased from Sigma Chemical Co. (St. Louis, United States). Media constituents, reagents used in electrophoresis and buffer components were purchased from Amersham Life Science (Little Chalfont, United Kingdom). All other reagents were of analytical grade.

### Fungal Strain and Growth Conditions

*Fonsecaea pedrosoi* (ATCC 46428, formerly 5VLP) isolated from a human patient with chromoblastomycosis (Oliveira et al., 1973) was used in all parts of the present work. Stock cultures were maintained on Sabouraud dextrose agar under mineral oil. The fungal cultures were kept at 4°C and transfers were made every 6 months. For sclerotic cell formation, cultures were incubated for 30 days under constant agitation (200 rpm) at 37°C in Erlenmeyer flask containing 100 mL of Butterfield’s chemically defined medium (pH 2.5): 5 mL glycerol, 0.1 g MgSO_4_, 1.8 g KH_2_PO_4_, 1.5 g NH_4_NO_3_, 5 mg biotin and 0.1 mg thiamine-HCl per liter (Alviano et al., 1992). For all the experiments, sclerotic cells were washed three times in saline (0.85% NaCl) and the number of cells was estimated by counting in a Neubauer chamber.

### Cell-Free Culture Supernatant and Protein Content

The sclerotic cultures were centrifuged (4000 × *g*, 10 min, 4°C) and the supernatants were filtered in a 0.22-μm membrane (Millipore). The cell-free culture supernatants were 100-fold concentrated in a 10,000 molecular weight cut-off Amicon micropartition system (Beverly, MA, United States) (Palmeira et al., 2008). Protein concentration was detected by the method described by Lowry et al. (1951), using BSA as standard.

### Proteolytic Activity Measurements

Extracellular proteolytic activity was measured spectrophotometrically according to the method described by Buroker-Kilgore and Wang (1993). Briefly, 20 μL of concentrated supernatant (equivalent to 10 μg of protein) were incubated in the absence (control) or in the presence of BSA substrate (0.5 mg*/*mL). Alternatively, the concentrated supernatant was added to different buffers, such as 10 mM sodium citrate (pH 2.0 – 4.0), 50 mM phosphate buffer (pH 5.0 – 8.0) or 20 mM glycine-NaOH (pH 9.0 – 10.0) to determine the optimum enzyme pH. In addition, the supernatant was incubated in the presence of classical PIs (10 mM PMSF, 10 μM E-64, 10 mM 1,10-phenanthroline and 10 μM pepstatin A) and HIV-PIs at 100 μM (saquinavir, indinavir, ritonavir and nelfinavir) in order to distinguish the peptidase enzyme class. After 1 h at 37°C, 100 μL of reaction mixtures were transferred to wells on a microtiter plate containing 50 μL of water and 100 μL of a Coomassie solution (0.025% Coomassie brilliant blue G-250, 11.75% ethanol and 21.25% phosphoric acid). After 10 min, to allow dye binding, the plate was read on a Molecular Devices Thermomax microplate reader at an absorbance of 595 nm. One unit of proteolytic activity was defined as the amount of enzyme that caused an increase of 0.001 in absorbance unit, under standard assay conditions (Palmeira et al., 2008).

### Soluble Proteins’ Cleavage Profiles

In this set of experiments, the concentrated supernatant (20 μL, which is equivalent to 10 μg of protein) obtained from sclerotic cells were incubated for 16 h at 37°C in the presence of the following proteinaceous substrates: BSA, HSA, FBN, LAM, and IgG. These proteins were diluted in 10 mM sodium citrate (pH 4.0) to obtain a final concentration of 5 μg*/*mL. Then, the reaction mixtures were added to 10 μL SDS–PAGE sample buffer supplemented with 5% β-mercaptoethanol, and boiled at 100°C for 5 min. The degradation protein profiles were analyzed using 10% SDS–PAGE. Electrophoresis was carried out at 4°C, at 120 V. Then, gels were stained with silver nitrate to evidence the protein cleavage profiles. Controls were made by replacing concentrated culture supernatants with the same volume of citrate buffer (Palmeira et al., 2006a).

### Influence of Aspartic PIs on *F. pedrosoi* Development

To test the possible involvement of aspartic peptidases on *F. pedrosoi* viability, different cellular densities (10^2^ – 10^6^ sclerotic cells) were resuspended in Butterfield’s chemically defined broth medium. Aliquots (100 μL) of this suspension were added to sterile microcentrifuge tubes and then complemented with 10 μM of pepstatin A. A control was made by replacing the PI with phosphate-buffered saline (PBS, pH 7.2). Alternatively, 10^5^ sclerotic cells were also treated with different pepstatin A concentrations (0.1, 1, 5, 10, and 20 μM) or HIV-PIs at 10 μM. The mixtures were incubated for 20 h at 37°C without agitation. The sclerotic cells were then harvested by centrifugation, washed twice with PBS and re-inoculated into solid medium without drugs, in order to measure the colony-forming units (CFU) (Palmeira et al., 2008). Methanol, the diluent of PIs, was also tested.

### Statistical Analysis

All the experiments were repeated at least three times. All the systems were performed in triplicate, and representative images of these experiments are shown. The data was analyzed statistically by Student’s *t-*test using EPI-INFO 6.04 (Database and Statistics Program for Public Health) computer software. *P-*values of 0.05 or less were considered statistically significant.

## Results and Discussion

### *Fonsecaea pedrosoi* Sclerotic Cells Secrete Acidic Peptidase

**FIGURE 1:**
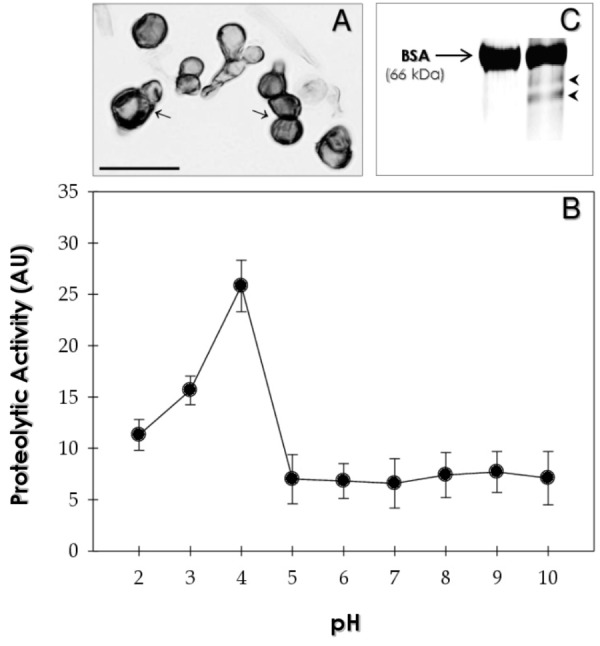
Peptidase activity released by *F. pedrosoi* sclerotic cells. **(A)** Optical microscopy showing the sclerotic cells obtained after *in vitro* growth in Butterfield’s chemically defined medium (pH 2.5). Dark-brown, thick-walled and spherical fungal cells exhibiting typical planate division can be observed (arrows). The image was captured by Zeiss Axioplan 2 microscope using an AxioCam camera. Bar: 20 μm. **(B)** The influence of pH on the peptidase activity released by sclerotic cells was evaluated. Fungal concentrated supernatant was incubated with the substrate (BSA) in different buffers: 10 mM sodium citrate (pH 2.0 – 4.0), 50 mM phosphate buffer (pH 5.0 – 8.0) or 20 mM glycine-NaOH (pH 9.0 – 10.0) for 1 h at 37°C. The reaction mixtures were measured at 595 nm and the proteolytic activity expressed in arbitrary units (AU), which was defined as the amount of enzyme that caused an increase of 0.001 in absorbance. **(C)** Substrate BSA was incubated for 1 h at 37°C in the absence (first slot) and presence of concentrated supernatant (second slot). Subsequently, both systems were supplemented with 10 mM sodium citrate pH 4.0 and subjected to SDS–PAGE. The arrow (on the left) shows the molecular mass of intact BSA. The arrowheads on the right indicate the fragmentation of BSA after proteolysis.

We have shown that chromoblastomycosis fungi secrete distinct peptidases and that these enzymatic profiles are closely related with fungal morphology and cultivation conditions (Palmeira et al., 2006a,b, 2008, 2017; Granato et al., 2015). Studies have reported that cell shape modifications are strategies used by different fungi to survive in the environment and inside the host (Wang and Lin, 2012). Considering that *F. pedrosoi* morphological transition from conidia/mycelia to sclerotic cells is an essential step to the establishment of chromoblastomycosis, the extracellular proteolytic profile of sclerotic cells was analyzed in this study. Thus, after the growth of *F. pedrosoi* sclerotic cells under chemically defined conditions (**Figure 1A**), the culture supernatant was incubated with soluble BSA, at different pHs (varying from 2.0 to 10.0), in order to evidence its possible cleavage by any released fungal peptidase (**Figure 1B**). The BSA degradation was observed only in acidic pH values, reaching a maximum hydrolytic activity at pH 4.0 (**Figure 1B**), which was clearly evidenced by the generation of polypeptide fragments with low molecular masses (**Figure 1C**). In the current study, we showed for the first time that sclerotic cells were able to produce an extracellular peptidase that was active at extremely acidic pH, as also described for conidial and mycelial forms of this fungus (Palmeira et al., 2006b, 2008), and for another chromoblastomycosis agent, *Phialophora verrucosa* (Granato et al., 2015). Coincidentally, filamentous forms of *F. pedrosoi* convert themselves into sclerotic cells in *in vitro* when incubated at acidic pH (2.5) (Mendoza et al., 1993). In addition, acidic peptidase produced by *F. pedrosoi* could facilitate its survival in acidic conditions usually detected inside the phagocytic cells (Palmeira et al., 2006b). Consequently, the differential pattern of peptidase expression may be essential for fungal adaptation to various environments, including host tissues (Naglik et al., 2003; Santos et al., 2007).

Recently, *F. pedrosoi* genome was entirely sequenced (Teixeira et al., 2017), which permits the beginning of the genomic analysis in order to better understand the functional organization of the genes and to decipher their potential roles. *Fonsecaea* species and other black fungi belonging to the bantiana-clade were predicted to produce a wide repertoire of different endo- and exopeptidases (Vicente et al., 2017). Peptidase-encoding genes were predicted using the MEROPS database, which revealed the abundance of three major classes: serine (S), metallo (M) and cysteine (C) peptidases. Members of *Herpotrichiellaceae*, which include *Fonsecaea*, presented specific and significant number of S09 (prolyl oligopeptidase), S33 (prolyl aminopeptidase) and M38 (isoaspartic dipeptidase) families (Teixeira et al., 2017). In that study, it was also detected an expansion of M38 proteins, which may be associated with β-aspartic dipeptidase that act in the release of iso-aspartate residues from peptides (Teixeira et al., 2017). Caspases, which are cysteine dependent aspartic-specific peptidase playing essential roles in programmed cell death and inflammation, occur in the *Fonsecaea* core genome (Madeo et al., 2002; Douglas et al., 2015; Vicente et al., 2017).

### The Acidic Peptidase Released by *F. pedrosoi* Sclerotic Cells Is an Aspartic-Type Peptidase

**FIGURE 2:**
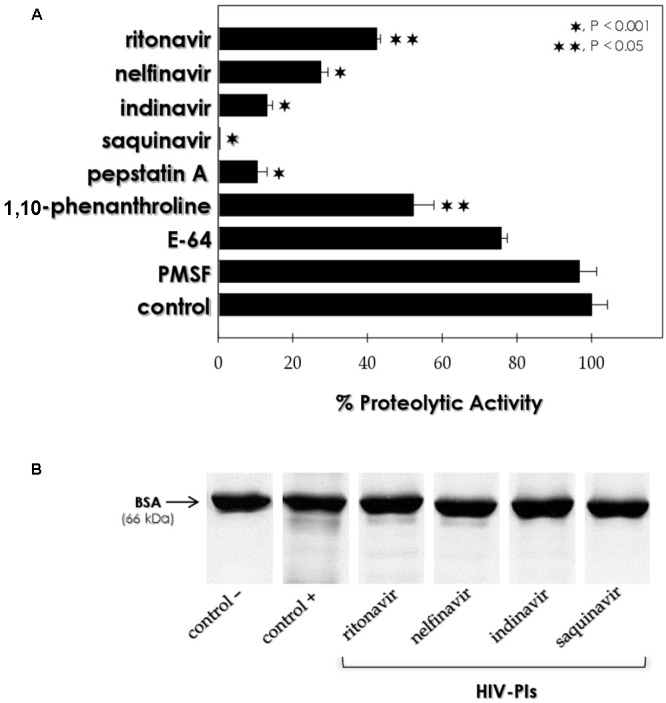
Effect of proteolytic inhibitors on the peptidase activity released by sclerotic cells. **(A)** Concentrated supernatant was incubated for 1 h at 37°C in 10 mM sodium citrate buffer, pH 4.0, and BSA, in the absence (control) or presence of classical proteolytic inhibitors, such as 10 μM pepstatin, 10 mM 1,10-phenanthroline, 10 μM E-64 or 10 mM PMSF; and 100 μM of the following HIV-PIs: ritonavir, nelfinavir, indinavir, or saquinavir. BSA supplemented exclusively with buffer was used as control. Peptidase activity was determined as described by Buroker-Kilgore and Wang (1993). The symbols denote the system treated with inhibitors that had a substrate hydrolysis rate significantly different from control (^∗^*P* < 0.001 and ^∗∗^*P* < 0.05; Student’s *t*-test). The peptidase activity was converted to percentage considering the control value as 100%. **(B)** Representative gels showing the intact BSA molecule diluted in sodium citrate buffer, pH 4.0 (control –), BSA degraded by the extracellular peptidase secreted by sclerotic cells (control + ) and the BSA cleavage inhibition by HIV-PIs at 100 μM.

The effect of classic PIs on the acidic peptidase released by *F. pedrosoi* sclerotic cells was evaluated. Pepstatin A, a classical aspartic PI, was able to drastically block the fungal released proteolytic activity by around 90% (**Figure 2**). In addition, 1,10-phenanthroline (a metallo-PI/chelating agent) partially reduced the enzymatic activity by approximately 45% (**Figure 2**). Conversely, sclerotic cells acidic peptidase activity was not significantly inhibited by PMSF (a serine PI) or E-64 (a cysteine PI). Taking into consideration the extremely acidic pH for peptidase activity as well as its inhibitory profile, the peptidase released by *F. pedrosoi* sclerotic cells can be classified as an aspartic-type peptidase. Our results corroborate previous *in silico* studies that predicted the presence of aspartic peptidase-encoding genes in *F. pedrosoi* genome (Teixeira et al., 2017; Vicente et al., 2017). Aspartic peptidases are characterized in different ways, according to their catalytic properties, cellular localization and pepstatin A inhibition, for example (Santos et al., 2013). Pepstatin A was also able to block the aspartic peptidase activities produced by other pathogenic filamentous fungi, including *Sporothrix schenckii, Aspergillus fumigatus, Paracoccidioides brasiliensis, Pseudallescheria boydii, Scedosporium aurantiacum*, and *Trichosporon asahii* (Tsuboi et al., 1988; Vickers et al., 2007; Tacco et al., 2009; Silva et al., 2012; Han et al., 2017; Valle et al., 2017). In order to confirm our results, the effects of four different HIV-PIs, which are capable of blocking the proteolytic enzymes belonging to the aspartic peptidase class, were tested on the fungal peptidase at a concentration of 100 μM. The HIV-PIs restrained the aspartic proteolytic activity from *F. pedrosoi* sclerotic cells as follows: saquinavir was the most effective, inhibiting the enzymatic activity in 99%, while indinavir, nelfinavir and ritonavir reduced the peptidase activity in 85, 70% and 55%, respectively (**Figure 2**).

Aspartic-type peptidases produced by several fungi were also inhibited by HIV-PIs (Santos et al., 2013). For instance, amprenavir was the most potent inhibitor of the secreted aspartic peptidases (Sap), the principal virulence factor produced by *Candida albicans* (Braga-Silva et al., 2010). This inhibitor reduced the activity of Sap2 by around 90% at 100 μM. Recently, Valle et al. (2017) reported that saquinavir and atazanavir were able to impair the aspartic peptidase activity secreted by *T. asahii* by around 50 and 70%, respectively. The HIV-PIs ritonavir, indinavir and nelfinavir also inhibited the aspartic peptidase activity produced by both conidial and mycelial forms of *F. pedrosoi* (Palmeira et al., 2006b, 2008), while saquinavir did not significantly alter the mycelial aspartic proteolytic activity (Palmeira et al., 2006b). These data indicated that *F. pedrosoi* morphotypes secrete distinct aspartic peptidase activities, which respond differently to the HIV-PIs tested (**Table 1**). Studies have reported the differential expression of aspartic peptidases during the fungal morphogenesis and that aspartic PIs might control this essential phenomenon to the establishment of fungal infection (Braga-Silva et al., 2010; Santos et al., 2013).

**Table 1:** Overview of the action of aspartic peptidase inhibitors on the peptidase activity and viability of *F. pedrosoi* morphotypes.

Fungal	Secreted aspartic peptidase		% of peptidase inhibition					% of growth inhibition^∗^				
Form	Optimum pH	Enzymatic activity (AU)	PEP	IDV	RTV	NFV	SQV	PEP	IDV	RTV	NFV	SQV
Conidia	4.0	42.7 ± 6.5	95	71	58	93	70	99	20	25	99	90
Mycelia	2.0	113.5 ± 8.1	95	97	97	90	28	ND	ND	ND	ND	ND
Sclerotic	4.0	26.0 ± 2.3	90	85	55	70	99	80	91	88	95	67

*The proteolytic activity and fungal growth inhibition were determined as detailed in the current material and methods and in the previous publications (Palmeira et al., 2006b, 2008, 2017). For peptidase activity, aspartic peptidase inhibitors, such as pepstatin A (PEP) at 10 μM and HIV-PIs at 100 μM [indinavir (IDV), ritonavir (RTV), nelfinavir (NFV) and saquinavir (SQV)], were added to reaction medium to calculate the inhibition rate. The enzymatic activity was expressed in arbitrary unit (AU, the enzyme amount capable of increasing 0.001 in absorbance) or in percentage considering the control value as 100%. ^∗^Different morphotypes (1 × 10^*3*^ cells) were treated with 10 μM PEPS. Sclerotic cells were treated with 10 μM HIV-PIs, while the treatment of conidia were performed with 100 μM HIV-PIs. After 20 h, the cells were subjected to CFU assay to measure the viable cells.*

### Cleavage of Different Proteinaceous Substrates

**FIGURE 3:**
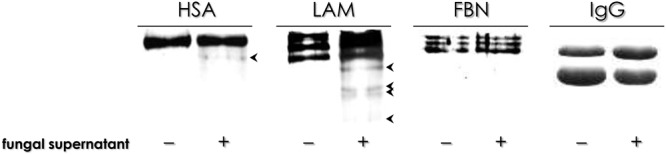
Cleavage of soluble proteinaceous substrates by aspartic peptidase activity released by sclerotic cells. The degradation profile was analyzed by 10% SDS–PAGE and the gels were stained with Coomassie brilliant blue R-250. ( + ) Concentrated culture supernatant from sclerotic cells incubated in the presence of human serum albumin (HSA), laminin (LAM), fibronectin (FBN), and immunoglobulin G (IgG) for 16 h at 37°C. (–) Control in which the proteinaceous substrates were supplemented only with PBS. The arrows on the right represent bands generated after substrates degradation.

To provide more information about the aspartic peptidase secreted by *F. pedrosoi* sclerotic cells, we tested its capability of cleaving key host proteinaceous substrates, including serum proteins (HSA and IgG) and extracellular matrix components (LAM and FBN). The results revealed that the aspartic peptidase of *F. pedrosoi* sclerotic cells was able to hydrolyze HSA and LAM, yielding low molecular mass polypeptides, while FBN and IgG were not degraded under the employed experimental conditions (**Figure 3**). Pepstatin A blocked the cleavage of both HSA and LAM (data not shown). The protein cleavage profiles detected herein are in contrast to those previously observed to aspartic peptidases produced by *F. pedrosoi* conidia and mycelia, since them were capable of hydrolyzing FBN and IgG (Palmeira et al., 2006a,b). The ability to cleave host structural proteins was also observed in other human pathogenic fungi; for instance, *A. fumigatus* secretes an aspartic peptidase capable of hydrolyzing elastin, laminin and collagen in the neutropenic mice lung (Lee and Kolattukudy, 1995). In addition, *C. albicans* cells produce Saps with wide substrate specificity that can cleave collagen and other host matrix proteins, which contribute to fungal virulence (Naglik et al., 2003; Singh et al., 2012). Thus, the degradation of host relevant proteins by fungal aspartic peptidases allow essential pathogenesis events such as dissemination and invasion of fungal cells, culminating in extensive damage and/or death of the host tissue (Monod et al., 2002; Naglik et al., 2003; Singh et al., 2012).

### Aspartic PIs Affect the Viability of *F. pedrosoi* Sclerotic Cells

**FIGURE 4:**
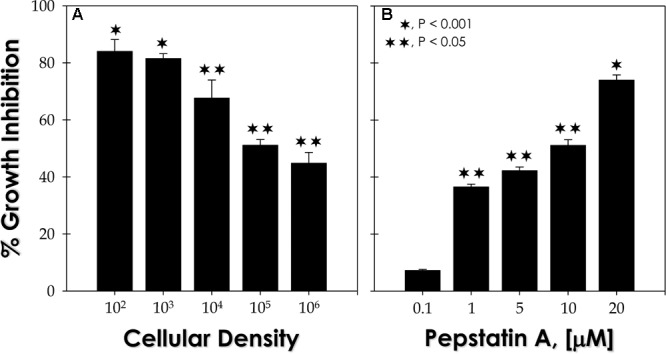
Effects of pepstatin A on sclerotic cell viability. **(A)** Different fungal densities (10^2^ – 10^6^) were treated with 10 μM pepstatin A. **(B)** sclerotic cells (10^5^) were treated for 20 h at 37°C with different concentrations of pepstatin A (0.1 – 20 μM). Inhibition ratio was determined by CFU assay and data represent the mean ± standard error of three independent experiments, which were performed in triplicate. The symbols denote the systems that had a growth rate significantly different from control (^∗^*P* < 0.001 and ^∗∗^*P* < 0.05; Student’s *t*-test).

**FIGURE 5:**
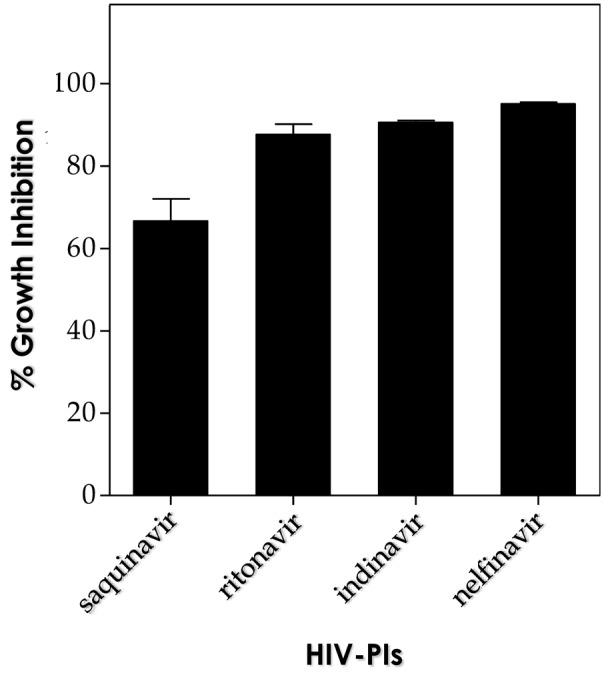
Effects of HIV-PIs on sclerotic cell viability. Sclerotic cells (10^5^) were treated for 20 h at 37°C with 10 μM of each HIV-PI (saquinavir, ritonavir, indinavir, and nelfinavir). Inhibition ratio was determined by CFU assay and data represent the mean ± standard error of three independent experiments, which were performed in triplicate. The symbols denote the systems that had a growth rate significantly different from control (^∗^*P* < 0.001 and ^∗∗^*P* < 0.05; Student’s *t*-test).

Based on the efficacy of pepstatin A in reducing the viability of both conidial and mycelial forms of *F. pedrosoi* (Palmeira et al., 2006a,b, 2008), herein, it was also investigated the effect of this classical aspartic-type PI on sclerotic cells. In this context, two relevant parameters were analyzed: distinct fungal densities (**Figure 4A**) and different concentrations of the PI (**Figure 4B**). Pepstatin A at 10 μM was able to considerably affect the viability of *F. pedrosoi* sclerotic cells in all the tested cellular densities (ranging from 10^2^ to 10^6^ fungal cells) (**Figure 4A**). In parallel, pepstatin A was also able to block the viability of sclerotic cells (10^5^ fungi) in a typically dose-dependent fashion (**Figure 4B**). Interestingly, the HIV-PIs at 10 μM were able to significantly arrest the fungal viability (>65%), when 10^3^ sclerotic cells were treated for 24 h, as follows: nelfinavir > indinavir > ritonavir > saquinavir (**Figure 5**).

Supporting these findings, several studies have reported that pepstatin A, as well as HIV-PIs have effective antifungal action in both *in vitro* and *in vivo* assays (Tsuboi et al., 1988; Borg-von Zepelin et al., 1999; Cassone et al., 1999; Monari et al., 2005; Consolaro et al., 2006; Cenci et al., 2008; Santos et al., 2013). For instance, pepstatin A exhibited an inhibitory effect on *C. albicans* growth, as well as reduced the adherence of *C. albicans* to vaginal mucosa epithelial cells by approximately 55% in asymptomatic, vulvovaginal candidiasis and recurrent vulvovaginal candidiasis patients (Tsuboi et al., 1988; Consolaro et al., 2006). The same inhibitor was able to suppress the proliferation of filamentous fungi as *S. schenckii* (Tsuboi et al., 1988). Moreover, HIV-PIs as ritonavir and indinavir exerted a therapeutic effect in an experimental model of vaginal candidiasis, with an efficacy comparable to the antifungal drug fluconazole (Cassone et al., 1999). Tipranavir showed anti-*C. neoformans* effect in an experimental assay, reducing fungal burden in the liver and brain of immunocompetent and immunodepressed mice (Cenci et al., 2008).

We have previously reported that aspartic PIs have direct antifungal action against conidia and mycelia of *F. pedrosoi* (Palmeira et al., 2006b, 2008). However, the mechanisms of action of these PIs have not been completely elucidated. Recently, we showed that HIV-PIs could affect essential virulence attributes expressed by *F. pedrosoi* conidial cells, such as surface molecules and extracellular enzymes, involved with chromoblastomycosis development (Palmeira et al., 2017). In this line of thinking, HIV-PIs treatment reduced mannose-rich glycoconjugates and melanin molecules, and increased glucosylceramides on the conidial surface of *F. pedrosoi*. The HIV-PIs were also able to inhibit the synthesis of both ergosterol and lanosterol as well as the secretion of aspartic peptidase, esterase and phospholipase by *F. pedrosoi* conidial cells (Palmeira et al., 2017). In the current study, we showed that pepstatin A was able to disturb sclerotic cells viability, varying according to fungal cell number and inhibitor concentration. Surprisingly, *F. pedrosoi* sclerotic cells, which are highly resistant to classic antifungals drugs, were more sensitive to pepstatin A than conidia (Palmeira et al., 2006b, 2017), since 10 μM of this inhibitor affected the cellular viability (inhibition of 50% of growth), even when 10^5^ and 10^6^ densities were used. In addition, the treatment of 10^5^ sclerotic cells with pepstatin A at 1 μM diminished fungal proliferation by around 40%. Therefore, it is important to consider that antimicrobial action is multifactorial, as it is dependent on the inoculum size and drug concentration (Gehrt et al., 1995; Palmeira et al., 2017). Moreover, antimicrobial activity can be microorganism- and/or morphology-dependent. Kasper et al. (2015) compared the clotrimazole antifungal action against *C. albicans* morphotypes and showed that hyphae were much more sensitive to this azole than the yeast form. In fact, studies have showed that fungal morphological changes are usually associated with intense modification of cell surface, physiology and immunology (Klein and Tebbets, 2007; Erwig and Gow, 2016).

## Conclusion

*Fonsecaea pedrosoi* causes chromoblastomycosis, an occupational and neglected disease difficult to treat using the current available therapies. In this context, fungal biology and physiology studies contribute to a better understanding of events related to this fungus pathogenicity, revealing key molecules to be attacked by antimicrobial agents. Altogether, our data showed the presence of aspartic-type peptidase in *F. pedrosoi* sclerotic cells and implied the involvement of this enzyme in the fungal viability. Results obtained in the current and previous studies (Palmeira et al., 2006b, 2008, 2017) revealed that morphotypes of *F. pedrosoi* can exhibit aspartic peptidase activities with distinct biochemical properties, including optimum pH, as well as different sensitivities to inhibitors, as summarized in **Table 1**. These findings indicate that differential expression of extracellular aspartic peptidases is directly dependent on the *F. pedrosoi* morphological stage, which can have an effect on the chromoblastomycosis pathogenesis. Studies have reported the fungal proteolytic enzymes detection may lead to a new inhibitors design to control diseases caused by these organisms (for review see, Santos et al., 2013). It is well-known that aspartic peptidases are essential targets of currently used drugs, as HIV-PIs, which are raising up like attractive candidates for antifungal therapies.

## Author Contributions

VP, LK, and AS conceived and designed the study. VP, FG, MG, and DA performed the experiments. DA, CA, LK, and AS contributed reagents, materials and/or analysis tools. VP, FG, MG, DA, CA, LK, and AS wrote and/or revised the paper. All authors analyzed the data.

## Conflict of Interest Statement

The authors declare that the research was conducted in the absence of any commercial or financial relationships that could be construed as a potential conflict of interest.
